# Optimal control of the spatial allocation of COVID-19 vaccines: Italy as a case study

**DOI:** 10.1371/journal.pcbi.1010237

**Published:** 2022-07-08

**Authors:** Joseph Chadi Lemaitre, Damiano Pasetto, Mario Zanon, Enrico Bertuzzo, Lorenzo Mari, Stefano Miccoli, Renato Casagrandi, Marino Gatto, Andrea Rinaldo

**Affiliations:** 1 Laboratory of Ecohydrology, École Polytechnique Fédérale de Lausanne, Lausanne, Switzerland; 2 Dipartimento di Scienze Ambientali, Informatica e Statistica, Università Ca’ Foscari Venezia, Venezia-Mestre, Italy; 3 Scuola IMT Alti Studi Lucca, Lucca, Italy; 4 Dipartimento di Elettronica, Informazione e Bioingegneria, Politecnico di Milano, Milan, Italy; 5 Dipartimento di Meccanica, Politecnico di Milano, Milan, Italy; 6 Dipartimento ICEA, Università di Padova, Padova, Italy; Rijksinstituut voor Volksgezondheid en Milieu, NETHERLANDS

## Abstract

While campaigns of vaccination against SARS-CoV-2 are underway across the world, communities face the challenge of a fair and effective distribution of a limited supply of doses. Current vaccine allocation strategies are based on criteria such as age or risk. In the light of strong spatial heterogeneities in disease history and transmission, we explore spatial allocation strategies as a complement to existing approaches. Given the practical constraints and complex epidemiological dynamics, designing effective vaccination strategies at a country scale is an intricate task. We propose a novel optimal control framework to derive the best possible vaccine allocation for given disease transmission projections and constraints on vaccine supply and distribution logistics. As a proof-of-concept, we couple our framework with an existing spatially explicit compartmental COVID-19 model tailored to the Italian geographic and epidemiological context. We optimize the vaccine allocation on scenarios of unfolding disease transmission across the 107 provinces of Italy, from January to April 2021. For each scenario, the optimal solution significantly outperforms alternative strategies that prioritize provinces based on incidence, population distribution, or prevalence of susceptibles. Our results suggest that the complex interplay between the mobility network and the spatial heterogeneities implies highly non-trivial prioritization strategies for effective vaccination campaigns. Our work demonstrates the potential of optimal control for complex and heterogeneous epidemiological landscapes at country, and possibly global, scales.

## Author summary

The development of vaccines has sparked high hopes towards the control of SARS-CoV-2 transmission without resorting to extensive community-wide restrictions. A fundamental unanswered question concerns the best possible allocation of a limited vaccine stock in space and time given a specific goal. We address this through an optimal control framework based on a reliable spatially explicit COVID-19 epidemiological model, where vaccine distribution is optimized under supply and deployment capacity constraints. This tool provides strategies for optimal allocations in different scenarios, yielding important improvements over considered alternatives. By accounting for the spatial heterogeneity and human mobility networks, the presented approach complements currently used allocation methods based on criteria such as age or risk.

## Introduction

Supply- or deployment-limited SARS-CoV-2 vaccines [1] pose the urgent question of a fair distribution of the available doses [2]. Current prioritization approaches typically target groups at higher risk of severe outcomes [3, 4], or their indirect protection by vaccinating those with higher disease transmission [3, 5, 6]. In this paper we will discuss how taking into account spatial heterogeneity in disease transmission when designing prioritization strategies significantly improves the effectiveness of vaccination campaigns. The distribution of doses inside each country is limited by the logistic capabilities of the healthcare network and the rate at which the vaccine stock is replenished. Decisions concerning the best allocation strategies are to be taken under these constraints. Moreover, both the complex coupling between regions due to human mobility and the spatial heterogeneity in disease history and control interventions make the discovery of such optimal allocation strategies an arduous task.

We propose an optimal control framework to explore COVID-19 vaccine distribution in space and time. We study the SARS-CoV-2 epidemic in Italy, where strong spatial effects arise from the geography of the disease, heterogeneous lockdown exit strategies, and post-lockdown control measures [7]. The optimal control framework is applied to a spatial model that has proved its reliability for Italy [8, 9], whose parameters are here sequentially updated through the assimilation of a year-long epidemiological record. This allows us to unravel the best possible vaccination strategy and probe the impact of vaccine allocations over the 107 Italian provinces.

The problem of vaccine allocation is of primary importance for public-health officials, epidemiologists, and economists [10, 11]. Roll-out strategies are conventionally based on the prioritization of individuals at risk, such as health workers and elderly people [12–15]. However, the heterogeneous ways in which different regions may be affected by each successive wave raise questions about spatial prioritization strategies. What is the best feasible spatial allocation, given supply and logistic constraints? Would that differ significantly from current non-spatially-optimized plans? Should vaccines be distributed based on demography or would it be better to prioritize areas currently subject to an outbreak? How relevant are the susceptibility profile and modeled future transmission in each region?

Epidemiological modeling has long been used to answer questions about the impact of vaccination campaigns, often by comparing outcomes under different scenarios [16, 17]. Optimization of epidemiological models, i.e, the search for the best possible course of action that maximizes or minimizes an objective metric, has been carried out theoretically since the seventies [18–20]. Recent dramatic improvements of both algorithms [21] and computational power prompted applied studies using different methods to rigorously find optimal mitigation strategies [22–24]: most of the time trough iterative parameter search [25, 26], but also using genetic algorithms [27], greedy algorithms [28] or solving the Hamilton-Jacobi-Bellman equations [29, 30].

Interesting developments have recently arose during the ongoing SARS-CoV-2 pandemic [13, 31, 32]. The urgency of effective vaccination campaigns led to the development of modeling frameworks for the optimization of vaccine allocation, based on age or risk [3, 4, 12, 13], space [33], dose timing [34, 35], and the deployment of testing resources, using optimal control [36] or Bayesian experimental design [37], along with prioritization based on social contact networks [38].

To the best of our knowledge, optimal spatial allocation of COVID-19 vaccines at a country scale has never been performed yet. This question is distinct from, and complementary to, risk-based prioritization. Spatial heterogeneities in disease transmission are complex, as seen during the initial outbreaks [8, 9, 39], supporting the significance of the posed problem towards an effective control of the epidemic. However, the connectivity network underlying spatial epidemiological models may generate complex large-scale control problems whose solution requires tailored formulations and efficient algorithms.

This work aims to find optimal strategies for this problem through state-of-the-art optimization methods based on distributed direct multiple shooting, automatic differentiation, and large-scale nonlinear programming [40–43]. This approach allows us to solve the large-scale optimization problems arising from epidemiological models, even when considering hundreds of spatial nodes.

## Materials and methods

The formulation of the optimal control problem has three main components: 1) an objective function to be minimized, here the number of new infections; 2) the spatial epidemiological model [8, 9] governing the transmission dynamics with the daily vaccination rates in each province as control variables; and 3) the set of constraints that the control must satisfy, in our case the limitations on vaccine administration rate in each province and the total vaccine stock in Italy.

**1) Objective function**. Optimizing calls for a metric, whose selection is critical in determining the optimal solution and its outcome. The choice of an objective function relates to health, economy, and ethics. Possible candidates are the minimization of, e.g., DALYs (the Disability-Adjusted Life Years), the number of deaths, disease exposure, or economic loss [44]. All these objectives are linked and may be combined. As the model considered for this work does not have risk classes, we optimize for the minimization of the incident infections in Italy from January 4, 2021, to April 4, 2021. Minimization of the deaths would yield the same results under the assumptions used in the model.

**Fig 1:**
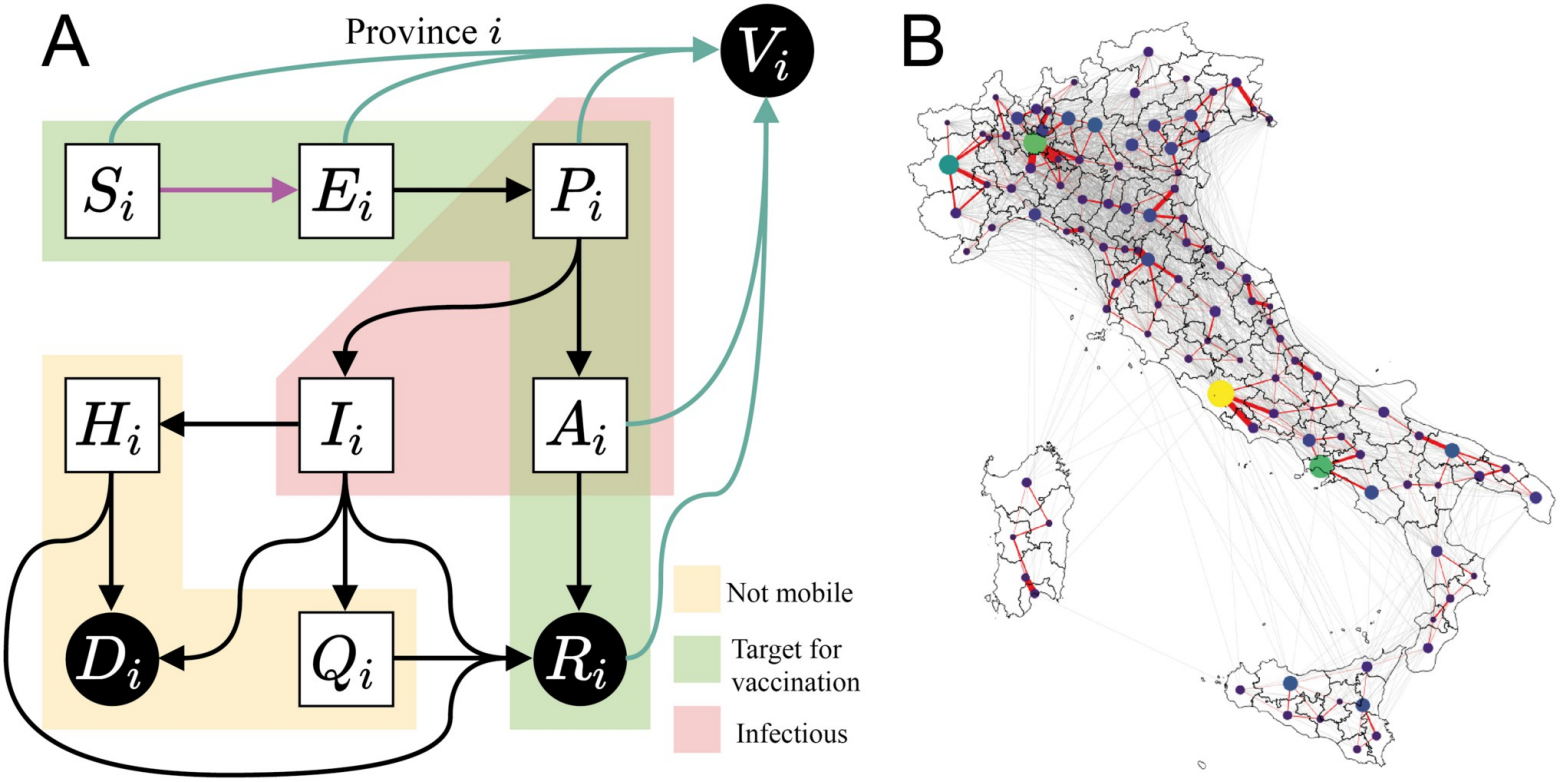
Model setup. (A) Diagram representing the compartments of the epidemiological model and the possible transitions in a single province. We control the vaccination rate (teal arrows), aiming at minimizing incident infections (pink arrow). Individuals in compartments outside of the yellow block are able to move along the mobility network shown in (B), hence the force of infection in a province is coupled with the dynamics of other connected provinces. To reduce the problem to a tractable size, we only consider the most important connections (red edges) when optimizing, but we use the full network (red and grey edges) to assess our strategies. A discussion on the effect of this simplification is provided in section C.2 in S1 Text. Nodes’ size and color display each province’s population, and edges’ width shows the strength of the coupling between a pair of provinces. Base map layer from the Istituto Nazionale di Statistica; Istat, istat.it, CC-BY 3.0.

**2) Epidemiological model**. Incidence and deaths are projected using the spatially distributed epidemiological model devised by Gatto et *al*. [8] and further improved by Bertuzzo et *al*. [9]. The model subdivides the Italian population into 107 provinces represented as a network of connected nodes. Each province has local dynamics describing the number of individuals present in each of the model compartments: susceptible *S*, exposed *E*, pre-symptomatic *P* (incubating infectious), symptomatic infectious *I*, asymptomatic infectious *A*, hospitalized *H*, quarantined *Q*, recovered *R*, and dead *D*. A tenth compartment, vaccinated individuals *V*, is added to the original nine, as shown in Fig 1A. Compartments *P*, *A*, and *I* have different degrees of infectiousness and contribute to the force of infection (Eq (C) and Eq (D) in S1 Text), which represents the rate at which susceptibles *S* become infected and enter the exposed compartment *E*.

Except for those in *H*, *Q*, *D*, or *I* states, a fraction of individuals commutes between provinces along the mobility network, thus we introduce node-to-node disease transmission along the network shown in Fig 1B. Thus, the force of infection in each province has a local and a mobile component. The local component describes transmission among the individuals that do not leave the province. The mobile component considers that local susceptibles may enter in contact with infected individuals that are traveling, and oppositely, susceptible commuters may become infected through contact with local infected. Connected provinces contribute to this process depending on the strength of the mobility fluxes from and to the node of interest. These mobility fluxes change in time due to the governmental policies introduced to reduce transmission among regions (complete model equations and more details about the data used to construct the mobility network and its use in the model are presented section B in S1 Text).

**Fig 2:**
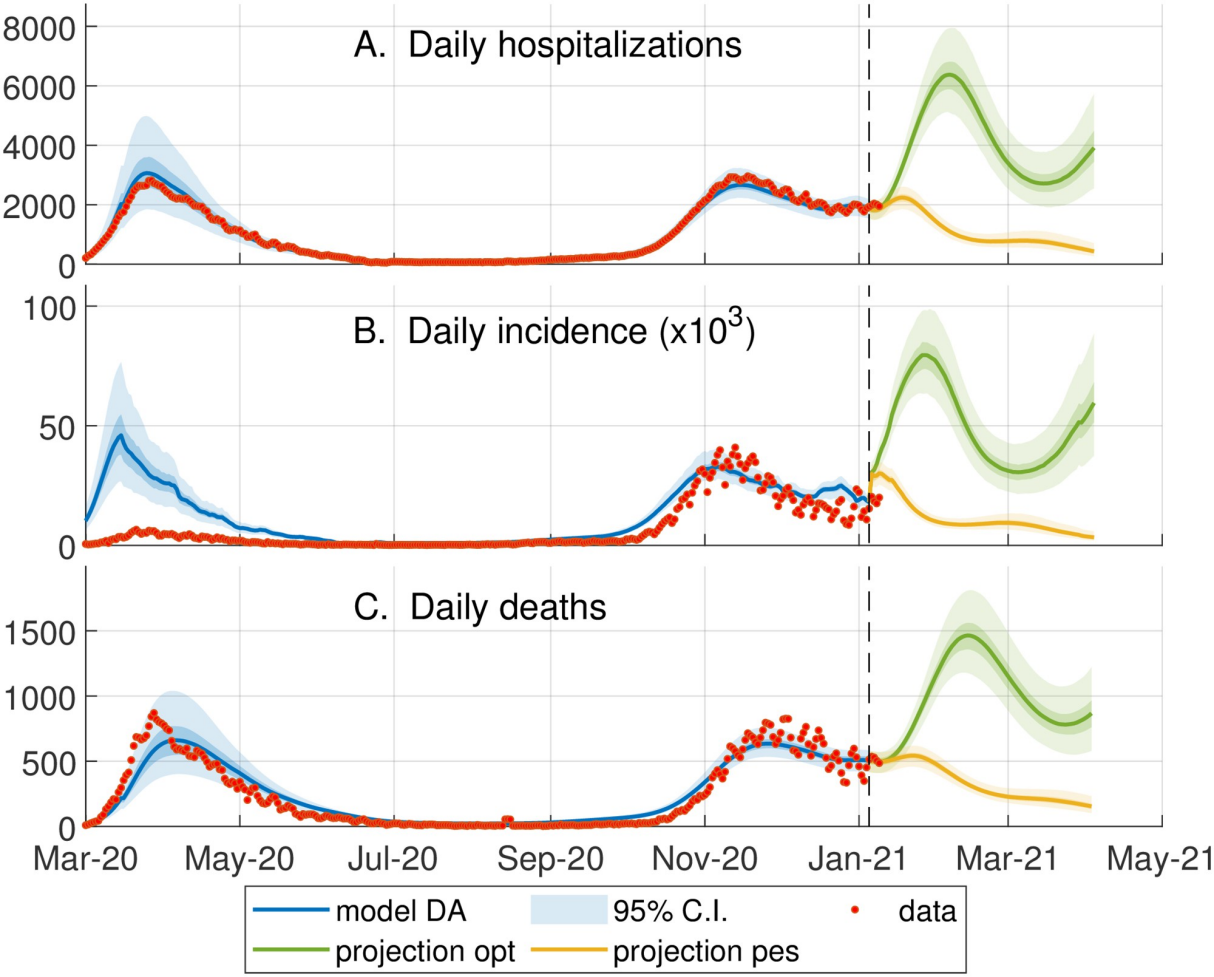
Data assimilation and scenarios for optimization. Comparison between the model outputs (95% confidence interval (CI) of the ensemble, blue shaded area) and the corresponding epidemiological data (red circles, obtained from the bulletins of the Dipartimento della Protezione Civile, https://github.com/pcm-dpc/COVID-19) from March 2020 to January 2021. The orange and green shaded areas respectively show the ensemble dynamics (95% CI) of what we called pessimistic and optimistic transmission scenarios from January to April. The optimal vaccination strategy in the optimistic (or pessimistic) scenario is computed for the the continuous green (or orange) line, representing the model trajectory obtained using the median of each ensemble parameter. (A) The data on the daily hospitalizations is estimated as described in [9]; this data at the regional level is assimilated on a moving window of 14 days to update the model parameters describing the local transmission rates (see section D in S1 Text). (B) Daily number of newly exposed individuals versus the reported positive cases. Note that the large discrepancy between model and data during the first wave is due to the low testing capacity at the beginning of the epidemic (C) Daily number of deaths.

The epidemiological model, previously calibrated during the first wave of COVID-19 in Italy [8, 9], is updated up to January 4, 2021 using an iterative particle filtering, which infers the regional transmission on a moving temporal window of two weeks. This data assimilation scheme allows us to capture the second wave of infections that hit Italy in the Fall of 2020, a necessary requirement to generate model projections that take into account the whole epidemic history, as shown in Fig 2. In our approach, model projections are described by an ensemble of a hundred trajectories associated with different parameters, whose distributions quantify the model uncertainty. We consider two projection scenarios characterized by two possible rates of epidemic transmission, see Fig 2. The “Optimistic” scenario assumes a constant lowering of transmission from January 4, 2021, to April 4, 2021; the “Pessimistic” scenario considers a gradual increase in transmission until mid-February 2021, which results in a third wave.

The control variable is the vaccination rate in each province. We assume one-dose vaccines with an instantaneous 100% efficacy, while in reality the vaccine efficacy and immunity duration depend on the vaccine type. As we focus on spatial patterns and differences among vaccination strategies for a given supply, this assumption does not affect the conclusion of our work. Finally, we impose that vaccine protection persists during the three months of projection considered.

For each scenario, the optimal control problem is solved for one reference model trajectory, whose parameters and state on January 4, 2021, are obtained as the median values of the 100 model realizations. In this way, the reference trajectory approximately represents the ensemble median in each province. Then, we assess the effectiveness of the optimal allocation on the full ensemble of trajectories.

**3) Constraints**. We define two types of constraints: supply constraints, which determine the weekly delivery to the national stockpile; and logistic constraints, which limit the maximum rate of local vaccine distribution in each province.

The supply constraints ensure that the model does not distribute more vaccines than what is actually available in stock. We assume that the national supply of vaccine doses is empty on January 4, 2021, and is replenished every Monday. We consider four scenarios with weekly deliveries of 125’00, 250’000, 479’700 (the latter is the most realistic and our baseline value), and 1M vaccine doses (additional results for scenarios with 1.5M and 2M doses delivered each week are shown in Table B and Fig I in S1 Text).

From the national stockpile, doses may be allocated to any of the 107 Italian provinces, but the logistic constraints limit the rate at which it is possible to distribute the vaccines in each province. We assume that the maximum number of individuals who can be vaccinated in a province per day is proportional to the province’s population, such that the national maximum distribution capacity equals 500’000 doses per day, i.e., 3.5M per week if every province vaccinates at its maximum rate (which in retrospect is close to Italy’s vaccination rate as of May 1, 2021).

The objective, the model, and the constraints may be tailored to specific applications within the proposed framework.

Using state-of-the-art large-scale nonlinear optimization solvers and automatic differentiation, we solve each scenario (optimistic and pessimistic, with different weekly stockpile deliveries) for the optimal vaccines allocation.

### Optimal control problem formulation

We provide a brief methodological description of the optimal control framework. The full equations are derived in the section C in S1 Text, along with implementation details and source code.

We denote *n* the number of spatial nodes (*n* = 107 provinces in Italy) and *m* the number of epidemic states in our model (*m* = 9 states). We denote as $x\left(t\right)\in R_{+}^{n\times m}$ the state of the system, i.e., *x*(*t*) = (*x*_1_(*t*), …, *x*_*n*_(*t*)) is a stack of vectors *x*_*i*_(*t*), each containing the epidemic variables *S*_*i*_(*t*), *E*_*i*_(*t*), *P*_*i*_(*t*), *I*_*i*_(*t*), *A*_*i*_(*t*), *Q*_*i*_(*t*), *H*_*i*_(*t*), *R*_*i*_(*t*), *V*_*i*_(*t*) for every province *i* = 1, …, *n*. We define $v\left(t\right)=\left(v_{1}\left(t\right),\ldots ,v_{n}\left(t\right)\right)\in R_{+}^{n}$, representing the rate of vaccine roll-out for every node *i* at time *t*, as our control variable. The epidemiological model can be described by the system of ordinary differential equations coupling disease transmission among all provinces shown in Eq (A) in S1 Text, and written compactly here as:

$$\begin{array}{c} \dot{x}\left(t\right)=F\left(x\left(t\right),v\left(t\right)\right) \end{array}$$ (1)

The national incidence, i.e., the sum of new infections in all provinces at time *t*, is selected as the running cost *L*(*x*(*t*), *v*(*t*)). Given our system with states *x* subject to the dynamics in Eq (1) and controls *v*, the optimal control problem is formalized as:

$$\underset{v(\cdot )}{\text{min}}\;\int_{0}^{T}L\left(x\left(t\right),v\left(t\right)\right)\;dt$$ (2a)

$$s.t.\;x\left(0\right)={\hat{x}}_{0},$$ (2b)

$$\dot{x}\left(t\right)=F\left(x\left(t\right),v\left(t\right)\right),\;\forall \;t\in [0,T],$$ (2c)

H(x(t),v(t))≤0,∀t∈[0,T], (2d)

where we aim at minimizing the cost function over the control horizon *T*, while enforcing the modeled SARS-CoV-2 transmission dynamics (Eqs (2b) and (2c)). Moreover, the constraints imposed by vaccine availability and the maximum vaccination rate are lumped in function *H* that expands to:

$$v_{i}\left(t\right)\geq 0,\;i\in I_{1}^{n},$$ (3a)

$$\int_{t_{d}}^{t_{d}+1}v_{i}\left(t\right)\;dt\leq v_{i}^{\text{max}}\propto N_{i},\;i\in I_{1}^{n},\;t_{d}\in I_{0}^{T},$$ (3b)

$$\int_{0}^{t}\sum_{i=1}^{n}v_{i}\left(t\right)\;dt\leq D(t),\;\forall \;t\in [0,T],$$ (3c)

where time is measured in days, and $I_{a}^{b}$ is the set of all integers *a* ≤ *k* ≤ *b*. Eq (3a) enforces that one can only distribute a non-negative amount of vaccine doses. Eq (3b) states the logistic constraints, which limit to $v_{i}^{\text{max}}$ the amount of individuals that can be vaccinated each day in each node; here *t*_d_ is the time at which each day starts. We impose that the daily vaccination capacity of each province is proportional to its population size *N*_*i*_, assuming a fair distribution of the sanitary infrastructure among provinces, as shown in the Fig A in S1 Text. The constraint on the national stockpile is expressed by Eq (3c), which ensures that the total vaccine allocation across all nodes does not exceed the stockpile *D*(*t*). The stockpile is replenished every Monday by the delivery of new vaccines, hence *D*(*t*) is a staircase function.

For an overview of the possible solution approaches for optimal control problems we refer the interested reader to [45, 46]. We solve the optimal control problem in Eq (2) by a direct method, also referred to as *first discretize, then optimize*, whose goal is to transform the control problem into a nonlinear programming problem. In particular, in this work, we use a variant of direct multiple shooting [40] tailored to distributed systems [41]. We split our time window [0, *T*] into *N* intervals [*t*_*k*_, *t*_*k*+1_], and we denote as *x*_*k*_ = *x*(*t*_*k*_) the states at time *t*_*k*_, and as *v*_*k*_ the controls in interval [*t*_*k*_, *t*_*k*+1_]. The continuous-time dynamics *F*(*x*(*t*), *v*(*t*)) in Eq (1) are transformed by numerical integration into the discrete-time model *f*(*x*_*k*_, *v*_*k*_). This discretization requires some care, and details are provided in section C in S1 Text. Finally, we obtain the following nonlinear programming problem:

$$\underset{x,v}{\text{min}}\;\sum_{k=0}^{N-1}l\left(x_{k},v_{k}\right)$$ (4a)

$$s.t.\;x_{0}={\hat{x}}_{0},$$ (4b)

$$x_{k+1}=f\left(x_{k},v_{k}\right),\;k\in I_{0}^{N-1},$$ (4c)

$$H(x_{k},v_{k})\leq 0,\;k\in I_{0}^{N-1}.$$ (4d)

Nonlinear programming problems may be solved by readily available solvers using, e.g., the primal-dual interior-point method. The main difficulty in solving the proposed nonlinear programming problem in Eq (4) is the large dimension of the system and the nonlinearity of the model. In order to bring the problem to a tractable form, we introduce three simplifications: (a) vaccines are administered instantaneously at the beginning of each day, rather than with a constant rate over the whole day; (b) the component of the force of infection taking into consideration the mobility of individuals across provinces is evaluated at the beginning of each day and remains constant through the day; and, (c) the mobility network is simplified, by keeping only the most important connections (see Fig 1), thus increasing the sparsity of the underlying spatial connectivity matrix. These simplifications deliver a significant computational advantage, and we verified that the impact on the model accuracy is limited. Note that, even though the optimal strategy is computed using the simplified model, its impact in terms of averted infections (shown in Results) is evaluated using the full epidemiological model without any of these simplifications. A more detailed discussion on this subject is provided in S1 Text.

The nonlinear programming problem arising from the simplified epidemiological model is nonconvex and involves approximately 10^5^ variables and slightly less than 10^5^ constraints. We formulate the problem using CasADi [42] and solve it using Ipopt [43] with sparsity-exploiting linear algebra solvers. In practice, solving a scenario of this optimal control problem takes between two to four days on a 36-cores 2.3 GHz CPU.

## Results

We obtain the optimal vaccination strategies for a set of eight scenarios drawn from the spatial model from January 4, 2021, to April 4, 2021. These scenarios are a combination of two projection scenarios (pessimistic vs optimistic) and four assumptions on the weekly stockpile delivery (125’000, 250’000, 479’700, or 1M doses delivered per week). In each scenario, the optimal solution is a spatially explicit vaccine roll-out policy, i.e. an indication of the number of vaccine doses to be deployed in each province each day.

### Performance of the optimal and alternatives vaccination strategies

Spatial prioritization based on epidemiological criteria, such as past [16] or future [17] incidence, has often been used in both real campaigns and prospective studies. In order to measure the improvements yielded by the optimal allocation strategy, we compare it against 12 alternative approaches which distribute the available weekly vaccine doses among provinces. These alternative strategies use an indicator variable to rank provinces, either i) their population; ii) the number of susceptible individuals (per inhabitant or absolute) at the beginning of the projection; iii) the future incidence as projected by the epidemiological model (per inhabitant or absolute) or iv) constant, equal for all provinces. The incidence indicator rankings are updated every day to reflect the change caused by past decisions. For each indicator we propose two variants: after ranking all the provinces, strategies either focus on the provinces where the indicator is the largest or allocate to all provinces proportionally to the indicator. Additionally, we further consider the greedy strategy presented in [28]. In the main text we present the results of the optimal strategy, the second-best strategy overall (indicator: incidence per inhabitant; allocation: focused), and proportional allocation strategies for incidence, susceptibility, and population. All other results, along with detailed pseudo-code for each strategy are presented in section E and F in S1 Text.

For each of the eight scenarios considered, we compute the number of averted infections with respect to a zero-vaccination baseline, and the number of averted infections per vaccination dose (see Table 1). In the optimistic transmission scenario, characterized by a recess of the epidemic, the vaccination campaign has a lower impact on the averted infections per dose as only a small percentage of the vaccinated individuals would have been at risk of transmission. As expected, the impact of the vaccination campaign is more evident in the pessimistic scenario where the optimal strategy averts up to 2.54 million infections given weekly stockpile deliveries of one million doses. By virtue of the law of diminishing returns, the number of averted infections per dose decreases (from 0.413 to 0.196) when increasing the weekly stockpile.

**Table 1:** Absolute number of averted infections (in millions) and averted infections per dose during the first three months of 2021 as evaluated for the reference trajectory (see Fig 2) for each strategy. The first column represents the considered scenarios of weekly stockpile replenishment, i.e., the number of doses delivered to Italy every week, ranging from 125’000 to one million.

Weekly stockpile delivery	Vaccination strategy	Averted infections (Millions)		Averted infections per dose	
		Optimistic	Pessimistic	Optimistic	Pessimistic
125’000	Optimal	0.146	0.672	0.0897	0.413
	Incidence per pop. (focused)	0.137	0.626	0.085	0.389
	Incidence per pop. (proportional)	0.106	0.509	0.0653	0.313
	Susceptible per pop. (proportional)	0.074	0.393	0.0456	0.242
	Population (proportional)	0.0691	0.387	0.0425	0.238
250’000	Optimal	0.228	1.100	0.0701	0.340
	Incidence per pop. (focused)	0.214	1.030	0.0666	0.321
	Incidence per pop. (proportional)	0.180	0.893	0.0554	0.275
	Susceptible per pop. (proportional)	0.139	0.734	0.0428	0.226
	Population (proportional)	0.132	0.735	0.0407	0.226
479’700	Optimal	0.334	1.700	0.0535	0.272
	Incidence per pop. (focused)	0.318	1.600	0.0515	0.259
	Incidence per pop. (proportional)	0.282	1.450	0.0452	0.232
	Susceptible per pop. (proportional)	0.240	1.280	0.0384	0.205
	Population (proportional)	0.232	1.290	0.0373	0.206
1M	Optimal	0.484	2.540	0.0372	0.196
	Incidence per pop. (focused)	0.467	2.440	0.0363	0.190
	Incidence per pop. (proportional)	0.437	2.310	0.0336	0.177
	Susceptible per pop. (proportional)	0.401	2.150	0.0309	0.165
	Population (proportional)	0.399	2.180	0.0307	0.168

**Fig 3:**
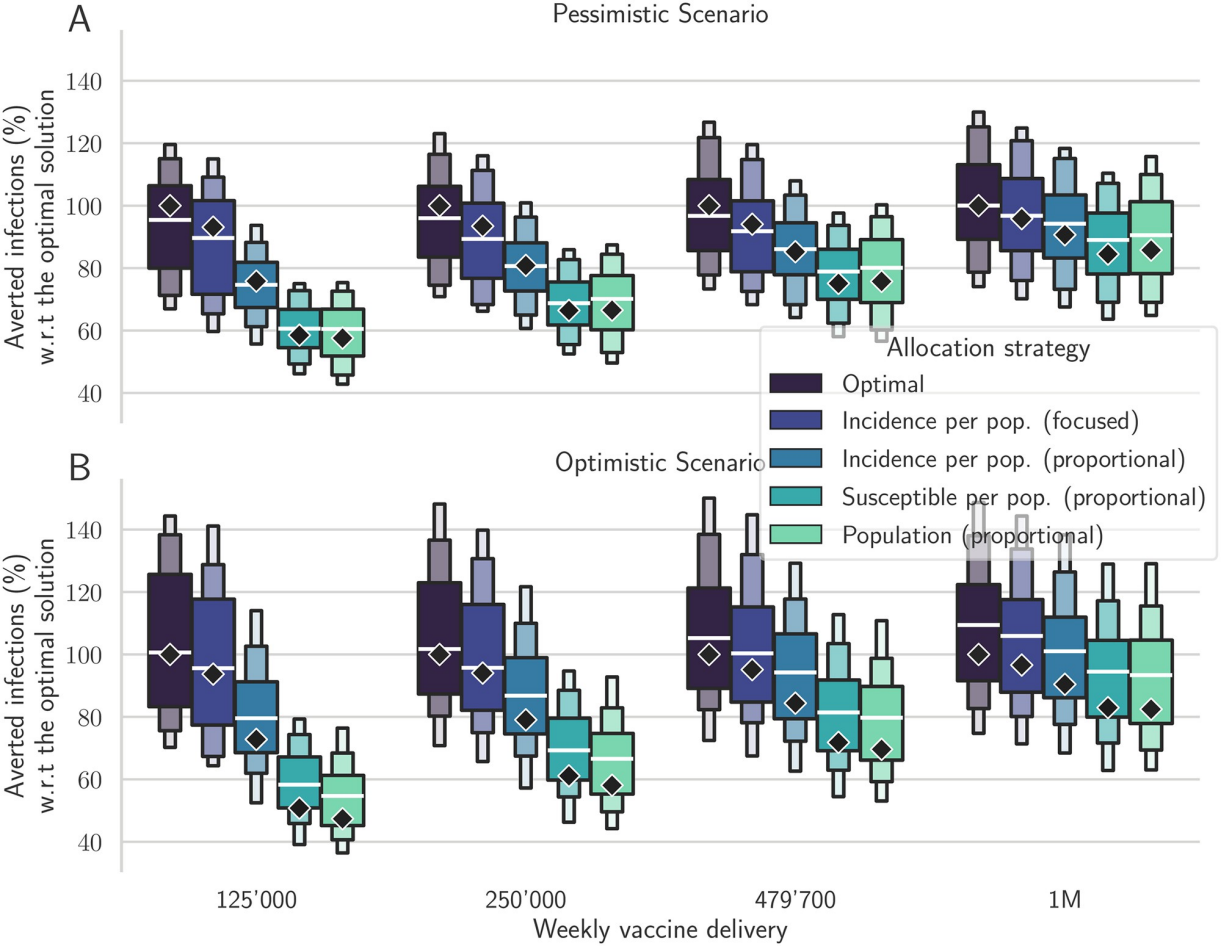
Comparison between different vaccine allocation strategies. Percentage of averted infections per vaccine dose from January 4, 2021, to April 4, 2021, resulting from province-scale vaccine allocation strategies for both the pessimistic (A) and the optimistic (B) scenarios based on the following vaccination strategies: the optimal solution, proportional to the province population, proportional to the susceptible individuals, proportional to the projected incidence, and focused on the provinces with the largest weekly incidence (see color codes in the legend). We optimize the vaccine allocation for the reference trajectory (the median trajectory in the model projections, indicated as diamonds in the figure), and assess the performance of the computed vaccination strategy over the whole posterior of trajectories (boxen plots). For each projection scenario, the results are normalized by the number of averted infections in the reference solution (see Table 1 for the absolute values). Results for alternative scenarios and vaccination strategies are shown in Fig I in S1 Text.

The optimal solution always outperforms all the explored alternative strategies in terms of the number of averted infections and in terms of averted infections per dose allocated (see Table 1, and Tables A and B in S1 Text). After extensive hyper-parameter tuning, the alternative strategy focusing on the provinces with the largest incidence has the closest results to the optimal strategy, with a difference of less than 10% in each scenario. Instead, the other strategies are significantly less effective. The improvement between optimal and incidence-based (proportional) allocation is significant, ranging from 9.0% (pessimistic, 1M doses/week) to 27.4% (optimistic, 125’000 doses/week). In Fig 3, the black diamonds represent the percentage of averted infections obtained using each strategy for the reference trajectory, normalized with respect to the averted infections resulting from the optimal strategy. We observe that, in both the optimistic and pessimistic scenarios, the optimal strategy has the largest relative benefits for the smallest stockpile.

In the pessimistic scenario (see Fig 3A), when 479’700 doses are available each week, the averted infections associated with the optimal strategy in the reference projection are 0.272 per dose: 24.6% more compared to the strategies based on population or susceptible distributions (0.205 averted infections per dose), and more than 14% higher compared to the strategy based on the projected incidence per inhabitant (0.232 averted infections per dose), while only 4% higher than the focused incidence strategy. These differences are smaller but still significant when increasing the weekly stockpile deliveries up to 1M doses; similar results are obtained also for the optimistic transmission scenario (Fig 3B).

We recall that the optimal control strategy is computed for a reference model trajectory, which is the median of an ensemble of 100 realizations. To further investigate the effectiveness of the optimal solution, we apply it to all trajectories of the ensemble. The box plots in Fig 3 display the main quantiles of the averted infections computed for the ensemble of trajectories. We observe that the optimal strategy still yields better results on the ensemble of projections compared to the other strategies, thus suggesting that the computed solution is robust even under the presence of perturbations in the forecasts of the epidemic dynamics. More importantly, for each realization of the ensemble and each projection scenario, the optimal strategy systematically averts more infections than any of the other control strategies.

The same conclusions hold while considering all alternative strategies (see Fig I and Table A and B in S1 Text). Finally, we present in Fig O in S1 Text, an additional sensitivity analysis where the epidemiological dynamics are shuffled between provinces, and thus are completely different from the projections used to design the strategies. This confirms that, despite its specialization, the optimal strategy does not under-perform with respect to alternatives in such scenarios.

Our results therefore suggest that it is possible to considerably increase the impact of vaccination campaigns by optimizing the vaccine allocation in space and time. For this task, optimal control provides the best possible strategy and sets a benchmark for the theoretical potential of a vaccination campaign.

### Analysis of the optimal vaccine allocation

The optimal vaccine allocation obtained as the solution of the optimal control framework is complex to analyze. We will attempt to unravel the mechanism behind its performance. The strategy must obey the imposed logistic and supply constraints: 1) The vaccine stockpile is replenished every Monday by a fixed amount of doses (e.g., 479’700 doses in the baseline scenario), and 2) the maximum possible distribution capacity per province is limited, proportionally to the province population, so that the number of doses distributed across the country can be of 0.5M per day at maximum (more details in Fig A in S1 Text).

**Fig 4:**
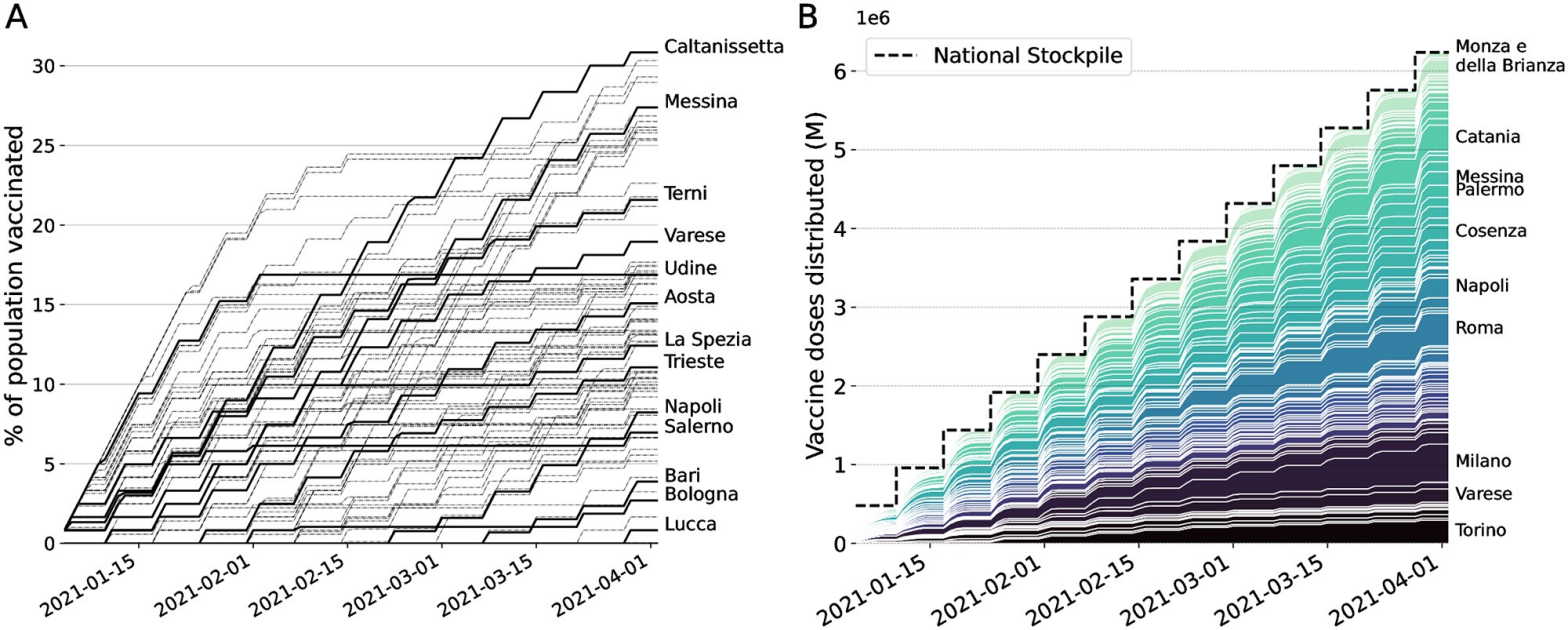
Optimal vaccine allocation for the baseline, pessimistic scenario. (A) Cumulative proportion of vaccine doses administered in the 107 provinces, some of which are highlighted. The local distribution rate is limited by a rate that is proportional to the population. This logistic constraint is visualized here as the maximum slope, equal for every province. (B) Stacked cumulative absolute number of vaccines in the 107 provinces of Italy. The national stockpile is shown in black and is replenished every week (on Mondays) with 479’700 doses. We display the name of the provinces with a final allocation of more than 150’000 doses.

We display the optimal vaccination strategy in time for 479’700 doses/week in the pessimistic scenario in Fig 4. We observe that the optimal allocation respects the two constraints on distribution (Fig 4A) and supply (Fig 4B). We observe that no province is vaccinated at the maximum possible rate during the whole campaign and that provinces display a variety of vaccination patterns. We also note that all vaccines received every Monday are always fully distributed during the following week, but that the rate of delivery on a national level increases with time (Fig 4B). Surprisingly, the optimal solution favors precise targeting over the speed of delivery, in order to allocate more doses to provinces where the impact of vaccines on the whole system is projected to be higher. Hence, in order to control infections, precise targeting may trump delivery speed.

**Fig 5:**
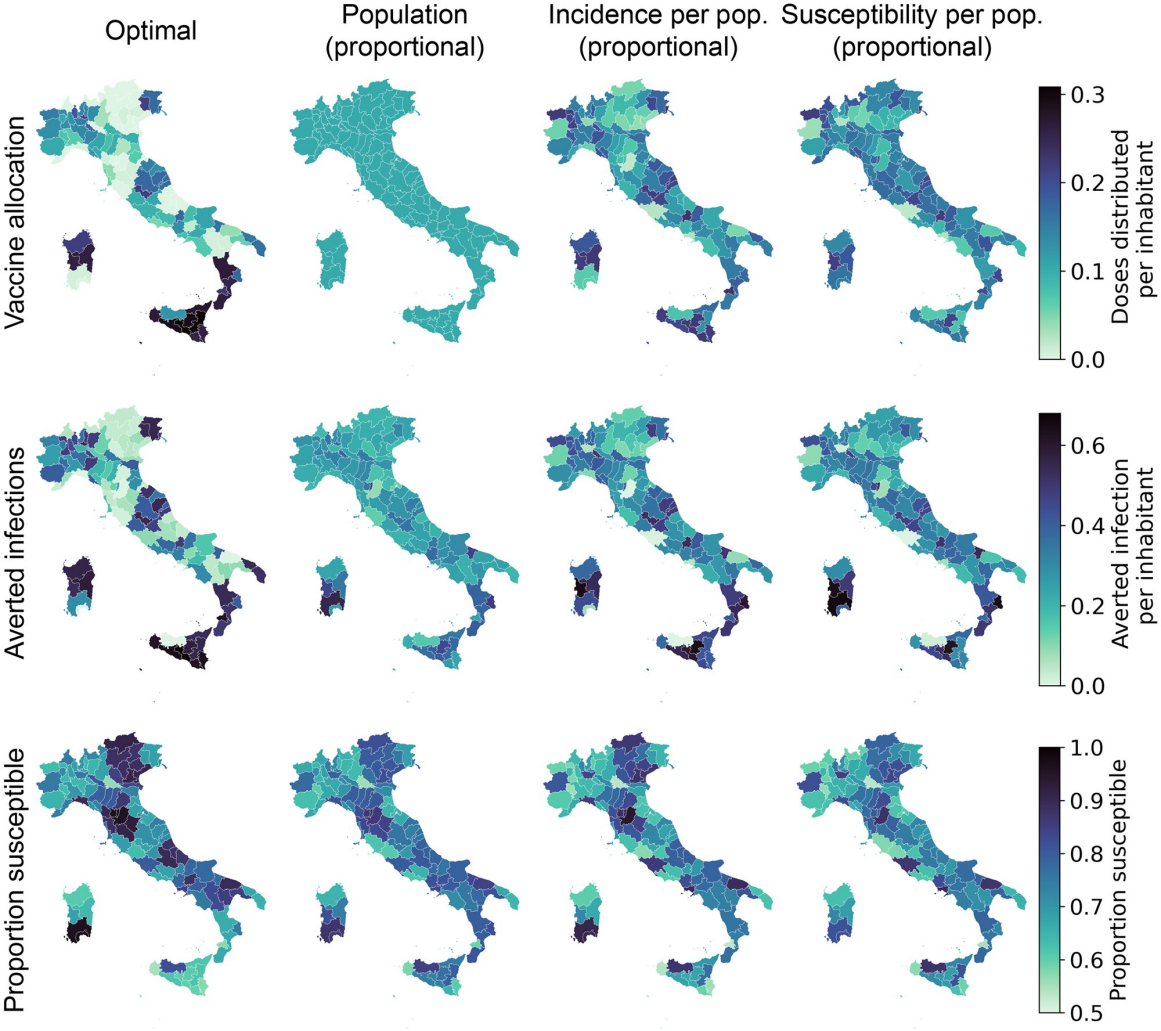
Spatial distribution patterns for the optimal allocation (left) and alternative strategies based on population, incidence, and susceptibility (additional alternative strategies are presented in section E and F in S1 Text). We show, for each province and strategy, the proportion of vaccinated individuals after the implementation of the strategy (top), the number of averted infections per inhabitant with respect to a no vaccination baseline (middle), and the proportion of individuals who are still susceptible at the end of the control horizon (bottom) for the pessimistic transmission scenario with a weekly stockpile delivery of 479’700 doses. Base map layer from the Istituto Nazionale di Statistica; Istat, istat.it, CC-BY 3.0.

Furthermore, we observe in the optimal solution that every time a province is vaccinated, the rate of vaccination is equal to the maximum rate allowed by the local logistic constraint, as it is the case for any focused alternative vaccination strategies. In Fig 5, one can already see by visual inspection that the optimal allocation distributes most of the available doses to a few provinces with high incidence. These provinces are neither the most connected nor the most populous in Italy. The optimal strategy makes then use of the information on the network connectivity to fine-tune the allocation and spreads the doses across more provinces than the incidence-based strategy.

**Fig 6:**
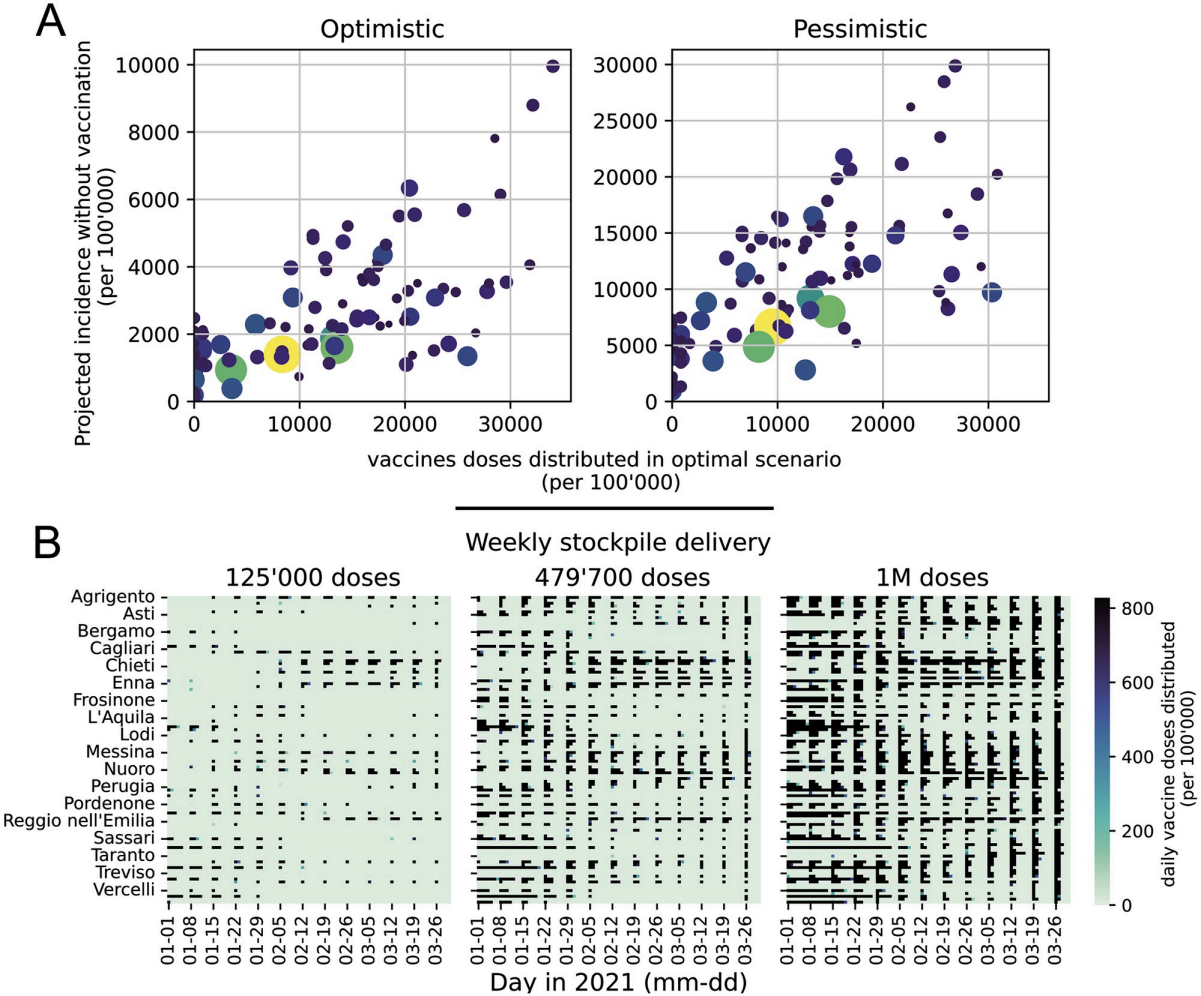
Analysis of the optimal solution. (A) Vaccinated population according to the optimal strategy against the projected incidence without vaccination, both normalized by province population and considering the scenario with a weekly stockpile delivery of 479’700 doses. (B) Heatmap of optimal allocation in space (y-axis, one square per province in alphabetical order) and time (x-axis, one square per day) for increasing weekly delivery scenarios (left to right). The color represents the proportion of individuals vaccinated on this day in this province by the optimal solution for the pessimistic transmission scenario; black is the maximum logistic rate per inhabitant (which is equal for all provinces). The same graphs for the optimistic transmission scenario and other weekly deliveries are shown in Figs L and M in S1 Text.

To further investigate these patterns, in Fig 6A we display the number of administered doses versus the incidence projected without vaccines (the proxy variables leading to the second-best control performance), both normalized according to the resident population in each province. We observe an allocation pattern whereby provinces with a higher incidence receive more vaccines, for both the pessimistic and optimistic scenarios. However, the allocation is nonlinear with respect to the projected incidence, suggesting that in order to better control the epidemic the optimal allocation strategy takes into account other factors such as the importance of each province within the mobility network, as well as the proportion of susceptibles. This complexity is further highlighted in the heatmaps in Fig 6B, where a complete picture of the spatio-temporal patterns is unraveled. For a stockpile delivery of 125’000 doses per week, we observe different patterns of local allocation in the provinces that receive vaccines. Along the course of the epidemic, the optimal strategy varies which groups of provinces are most vaccinated. When the weekly stockpile delivery is increased, we observe that the optimal solution allocates new doses by both further reinforcing already prioritized provinces (acting as a focused strategy) and by vaccinating new provinces (acting as a proportional strategy). The ability to allocate each dose where it is the most efficient, without tuning any hyper-parameter (such as a threshold when to act proportional versus focused) is a major benefit of the optimal control strategy. Additional analyses are provided in Figs J–N in S1 Text.

## Discussion and conclusions

Without any supply constraint, each country would vaccinate its population as fast as possible according to the available infrastructure. However, limitations in vaccine supply and rate of delivery are a reality for every country, hence the available doses should be deployed in space and time following a fair and effective strategy.

In stockpile-limited settings, like most current vaccination campaigns worldwide, careful allocation may significantly increase the number of averted infections and deaths. The goal is to distribute the vaccines where they have the strongest beneficial impact on the dynamics of the epidemic. However, designing an algorithm capable of computing spatially optimal allocation strategies in real heterogeneous settings is far from trivial and our approach is, to the best of our knowledge, the first attempt in this direction.

We developed a novel optimal control framework that delivers the best vaccination strategy under realistic supply and logistics constraints. This allows us to compute the allocation strategy that maximizes the number of averted infections during a projection of the COVID-19 epidemic in Italy from January 4, 2021, to April 4, 2021. Our results show that the optimal strategy has a complex structure that mainly reflects the projected incidence of each province, but also takes into account the spatial connectivity provided by the mobility network and the landscape of acquired population immunity. Although the reason why this strategy is optimal is not immediately intuitive, our simulations clearly outline that it significantly outperforms other, more straightforward strategies. This comparison suggests that the simplicity underlying intuitive vaccination strategies may undermine their effectiveness, and calls for complementing these simple approaches with rigorous and objective mathematical tools, such as optimal control, that fully account for the complexity of the problem.

With the present work, we showed that it is possible to solve optimal control problems for spatially explicit dynamical models of infectious diseases at a national scale, thus overcoming the computational limitations that, up to now, precluded this kind of applications. The proposed framework can account for any compartmental epidemic model, with up to hundreds of connected spatial nodes. Supply and logistic constraints can be adapted to the actual landscape of decisions faced by the stakeholders, such as no/reduced vaccine delivery on weekends, or the need for fairness in vaccine distribution, e.g., by ensuring that each province receives at least a fixed fraction of the available vaccines. This is especially important as in the optimal allocation some provinces might receive no vaccine at all. Moreover, while we assumed single-dose vaccines, one could optimize the timing between doses of multi-dose vaccines, in addition to every other control, in the same framework.

The present work is not devoid of limitations. The main one is that the optimal vaccination strategy strongly depends on the projection of the underlying epidemiological model. These projections, as every epidemiological projection, are subject to several sources of uncertainty, especially for long horizons, e.g., due to model design and calibration [47], assumptions about future events in transmission scenarios, and unforeseen events that may change the course of the epidemic (such as the importation of cases, the emergence of new virus variants, changes in disease awareness or social distancing policies). These aspects have an impact on the optimal vaccination strategy, which is reliable only if the projections given by the underlying model dynamics are sufficiently accurate. A successful approach developed by the automatic control community to tackle that issue, named Model Predictive Control [48], consists in compensating for the performance losses expected over long horizons by constantly adapting the optimal strategy. In this context, Model Predictive Control can be implemented using the following steps: (a) at the beginning of each week, the state of the system is estimated by using newly acquired epidemiological data; (b) the optimization problem is solved over a fixed prediction horizon using the estimated state as an initial condition; (c) the optimal strategy for the first week is applied and, as soon as the next week starts, these steps are repeated starting from (a). This method corrects the model inaccuracies by constantly resetting the initial state to the estimated one. Additionally, constraints may be updated to account for unexpected deliveries or new orders. Future work will aim at further evaluating the benefits of implementing this scheme for the design of optimal vaccination strategies. Stochastic control is another possible direction of improvement: the presented method solves the optimal control problem for the median trajectory of an ensemble of projections, but it is theoretically possible to compute an optimal vaccine allocation that accounts for the whole uncertainty range—either optimizing for the best expected performance or the best worst-case performance. However, methods for robust or stochastic control, that entail feeding the whole posterior distribution of trajectories into the optimal control framework, are most likely not computationally tractable for our problem. Instead, we provide the evaluation across the whole posterior of the optimal solution for the median trajectory, and find that its performance remains superior to the one of alternative strategies. We observe that for our problem, this method is enough to outperform other strategies. Moreover, the sensitivity analysis provided in section H in S1 Text demonstrates that despite its specialization, the optimal allocation strategy does not perform comparatively poorly when the model projections are inaccurate.

The epidemiological model underlying our control optimization has known validity and limitations [8, 9]. A significant simplification is the use of Google Community Mobility Reports (see section D in S1 Text) to estimate the variations in mobility across provinces and as a proxy for changes in social contacts. This approach does not explicitly account for complex social interactions and interventions (such as opening/closure of schools, shopping, mask-wearing, and social distancing), and data assimilation is necessary to adjust modeled transmission to the observed one. An additional limitation of the model for the specific scopes of this work is that it does not account explicitly for risk-based classes, and thus does not account for the heterogeneity that may result from the demography of the population, as well as from the age-related transmission and clinical characteristics associated with COVID-19. While surely limiting for operational use of the tools, we note that the scope of this paper is to provide a proof of concept of the relevance of spatial effects, which have not been addressed so far in the literature. To that end, we are confident that our results support the relevance of the research question posed. Our framework can be extended to optimize across both spatial and risk heterogeneity, provided that sufficient computational capacities are available to solve the resulting optimal control problem.

A counter-factual assumption in this work is that we consider a one-dose vaccine with full and instantaneous efficacy against transmission. At the time of development, the details about COVID-19 vaccines were not released, and this hypothesis allowed us to demonstrate our framework in a simple setting. Our framework can be further extended to account also for the simultaneous deployment of different vaccine types, some of which may require the administration of two doses. This extension is the subject of ongoing research, in particular to extend the modeling tools described here to accommodate the peculiarities of each authorized vaccine candidate while designing effective spatio-temporal deployment strategies.

In conclusion, in this work we presented and analyzed the optimal allocations of vaccines against SARS-CoV-2 at a country scale under different scenarios of epidemic transmission and vaccine availability. We designed a general optimal control pipeline that performs large-scale nonlinear optimization of epidemiological controls. We demonstrated our framework by coupling it with an existing model of COVID-19 transmission over the 107 provinces of Italy. This model was updated with a data assimilation scheme to reflect the epidemic state and history as of January 2021. Within our optimal control framework, we discretized, transformed and simplified the model to find the best possible allocation of vaccines under realistic stockpiles and logistic constraints. The optimal solution outperforms other strategies by a significant margin and proves robust across the uncertainty of the underlying model. As such, besides inherent limitations, it provides a benchmark against which other, possibly simpler vaccine rollout strategies can be usefully compared. We analyzed the mechanisms behind our optimal allocation and concluded that the complex interplay of spatial heterogeneity and human mobility requires non-trivial prioritization strategies in order to achieve the maximum effectiveness.

## Supporting information

S1 TextPresents the detailed optimal control methods, the description of all the alternative strategies, an in-depth analysis of the results and some additional results and figures.**Table A**: Absolute number of averted infections for the scenarios with the lower weekly stockpile delivery. **Table B**: Absolute number of averted infections for the scenarios with the largest weekly stockpile deliveries. **Fig A**: Local maximum vaccination rate $v_{i}^{\text{max}}$ for each province. This logistic constraint bounds the maximum number of vaccines to 0.5M of doses per day, with a local rate that is proportional to the node population. Here we show the maximum vaccination rate for each province (the constraint the solution has to comply with), in red, and the maximum rate prescribed by the optimal solution while simulating the pessimistic scenario with a stockpile delivery of 479’700 doses, in black. The optimal solution uses the maximal capacity of the logistic network, while respecting the constraint. **Fig B**: Comparison between the incidence in the exposed compartment *E* (per 1’000 people) as evaluated by the model simplified for the optimal control (red) and the full epidemiological model (black). Results for the pessimistic scenario without vaccination. The exposed compartment is very sensitive and exhibits the largest error among all compartments. In spite of this, the error is very small, justifying the simplifications undertaken. **Fig C**: Simplification of the mobility matrix to obtain a sparse and tractable problem for the optimal control framework. Note that, after computing the optimal vaccination strategy, we assess its effectiveness of on the full epidemiological model. Base map layer from the Istituto Nazionale di Statistica; Istat, istat.it, CC-BY 3.0. **Fig D**: Age-stratified outputs. Results of the post-processing algorithm for the computation of susceptibles (panel A), exposed (panel B) and deaths (panel C) among the five considered age classes. The algorithm provides results at the province level, which are here aggregated at the national level (see the section on age structure). **Fig E**: Modeled daily hospitalizations (blue) versus hospitalization data (red dots), regional detail of Fig 2A in the main text. The optimistic and pessimistic transmission scenarios are represented in green and yellow, respectively. **Fig F**: Modeled daily incidence (blue) versus the daily reported cases (red dots), regional detail of Fig 2B in the main text. The optimistic and pessimistic transmission scenarios are represented in green and yellow, respectively. **Fig G**: Values of the transmission parameters *β*_*i*_(*t*) in Eq (F) as estimated in the data assimilation procedure (blue). The values used in the optimistic and pessimistic transmission scenarios are represented in green and yellow, respectively. The red lines represent the reduction in mobility and transmission computed using google mobility data (coefficient 1 + *g*_*i*_(*t*)/100 in Eq (F)). **Fig H**: Projected incidence into the exposed compartment *E* (per 1’000 people) for the pessimistic (red) and optimistic (blue) scenarios. **Fig I**: Comparison of different allocation strategies. Percentages of averted infections per vaccine dose from January 4, 2021 to April 4, 2021 using different vaccine distribution strategies for the pessimistic (panel A) and the optimistic (panel B) scenario based on: the optimal solution, the spatial distribution of the population, the amount of susceptible individuals at the beginning of the vaccination campaign, and the projected disease incidence in the absence of control. We optimize a median realization of the modeled posterior (diamonds), and assess the performance on the whole posterior (box plots). The results are normalized by the number of averted infections in the optimized solution (see Tables A–B for absolute values). **Fig J**: Control and covariates for the optimistic scenario with a stockpile delivery of 479’700 vaccine doses. **Fig K**: Control and covariates for the pessimistic scenario with a stockpile delivery of 479’700 vaccine doses. **Fig L**: Vaccinated population according to the optimal strategy against the projected incidence without vaccination, both normalized by province population. Each dot represents a province, and the dot size is proportional to the population, while each symbol represents a weekly stockpile replenishment scenario. This corresponds to main text Fig 6A, but considering all four scenarios of weekly stockpile replenishment. **Fig M**: Time allocation for the pessimistic scenario with a stockpile delivery of 479’700. We see for each week, how the 479’700 doses are spread across the provinces, as a percentage. This view unravels the temporal pattern in the allocation. **Fig N**: Heatmap showing the allocation in space and time for different weekly delivery scenarios (left to right) and different transmission scenarios (Optimistic at the top, Pessimistic at the bottom). The x-axis represents time (one square per day) and the y-axis space (one square per province, in alphabetical order), and the color represents the proportion of individuals vaccinated on every day in each province by the optimal solution, with black displaying the maximum logistic rate per inhabitant, which is equal for all provinces. **Fig O**: Sensitivity analysis of different allocation strategies. Percentages of averted infections per vaccine dose from January 4, 2021 to April 4, 2021 using different vaccine distribution strategies for the pessimistic (panel A) and the optimistic (panel B) scenario for all alternative strategies. Here the median realization of the modeled posterior is optimized (diamonds), and the comparison is done on shuffled dynamics (random allocation of each province’s dynamics to another province, box plots). The results are normalized by the number of averted infections in the optimized solution (see Table A–B for absolute values).(PDF)
